# Comment on “South Vietnamese Rural Mothers' Knowledge, Attitude, and Practice in Child Health Care”

**DOI:** 10.1155/2016/7894746

**Published:** 2016-07-19

**Authors:** Madhavi Bhargava

**Affiliations:** Department of Community Medicine, Yenepoya Medical College, Yenepoya University, Mangalore 575018, India

The paper by Thac et al. is an important paper from the point of view of health care providers and policy makers in the context of Integrated Management of Childhood Illnesses (IMCI) in developing countries like India [1].

IMCI is an integrated approach which includes three main components [2]:

- Improving case management skills of health-care staff.
- Improving overall health systems.
- Improving family and community health practices.

The paper highlights the importance of the third and valuable component of IMCI, that is, “improving family and community health practices” by health education of mothers of under-five children. The figures mentioned in the paper clearly show that it is successful in achieving this third component.

India has made notable strides in tackling neonatal and childhood illnesses through a number of initiatives. These are in the form of addition of neonatal component in IMCI (IMNCI), training of community level grass-root workers such as Accredited Social Health Activists (ASHA) and Anganwadi Workers (AWW), Home Based Neonatal Care (HBNC), facility based IMNCI, infant and young child feeding practices (IYCF), and Rashtriya Bal Swasthya Karyakram (RBSK) to name a few [3].

Health education and training of grass-root workers are being done in India, but organized component of health education of mothers needs to be incorporated for improving health practices at family and community level. This can be systematically introduced at national level using the community based platforms of Village Health Sanitation and Nutrition Committees (VHSNC) and Mahila Arogya Samitis (MAS) in India.
